# Starch-Based Eco-Friendly Electrolyte from *Manihot esculenta* for the Anodic Synthesis of Nanostructured TiO_2_ Films

**DOI:** 10.1021/acsomega.5c11864

**Published:** 2026-04-30

**Authors:** Isabelli C. Baradel, Anna P. Simon, Tatiane L. C. Oldoni, Fauze J. Anaissi, Mariana S. Sikora

**Affiliations:** † Department of Chemistry, 74354 Universidade Tecnológica Federal Do Paraná (UTFPR), Campus Pato Branco, Via do Conhecimento, Pato Branco 85503-390, Paraná, Brazil; ‡ Federal Institute of Paraná (IFPR), Rodovia BR 163, 2115, Industrial 85700-000, Barracão, Brazil; § Department of Chemistry, Midwestern Paraná State University (UNICENTRO), Campus CEDETEG, Alameda Élio Antonio Dalla Vecchia, Guarapuava 85040-167, Paraná, Brazil

## Abstract

Developing sustainable synthesis routes for biomedical coatings is crucial to reducing toxic waste and minimizing the environmental footprint of implantable devices. Titanium dioxide (TiO_2_) nanostructured films, widely investigated for biomedical and environmental applications due to their biocompatibility, high surface area, corrosion resistance, and catalytic properties, are typically synthesized using ethylene glycol-based electrolytes (EBE). However, the toxicity and persistence of ethylene glycol (EG) raise significant environmental and safety concerns. This study introduced a greener alternative to conventional EBE by employing eco-friendly colloidal suspensions of *Manihot esculenta* starch. The environmental performance of the proposed electrolyte was quantitatively assessed using AGREEprep metrics, yielding a significantly higher greenness score (0.56) compared to EBE (0.29). Structural and morphological analyses by FE-SEM, XRD, and Raman spectroscopy confirmed the successful formation of nanoporous TiO_2_ films, with electrolyte composition playing a decisive role in pore organization. Subsequent thermal annealing promoted the crystallization of the initially amorphous oxide into the anatase phase, a feature desirable for biomedical applications. Electrochemical testing in artificial saliva demonstrated that the corrosion resistance of starch-derived TiO_2_ films can be tuned by starch concentration, reaching values comparable to or exceeding those obtained with EBE. These results highlight the feasibility and advantages of starch-based electrolytes, establishing a sustainable and practical pathway for fabricating TiO_2_ films tailored for biomedical applications.

## Graphical abstract

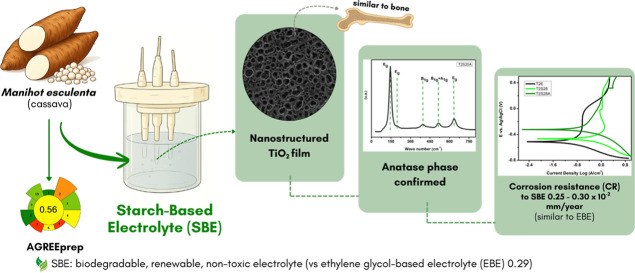

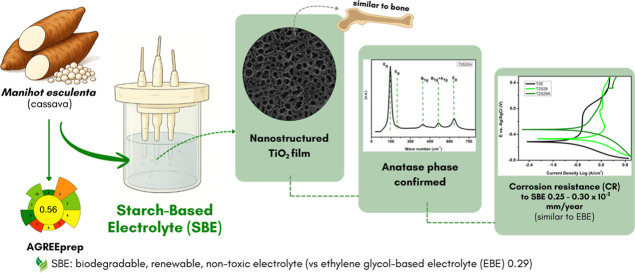

## 1 Introduction

Green chemistry has driven the urgent replacement of hazardous substances used in industrial processes with safer and more sustainable alternatives. Ethylene glycol (C_2_H_6_O_2_) is a colorless, sweet-tasting alcohol used in household and industrial solvents, mainly as an antifreeze agent, coolant, or raw material for polyester production.^1,2^ Exposure to ethylene glycol (EG) can lead to varying toxicity due to its rapid absorption through the skin, respiratory tract, or gastrointestinal tract. This exposure may result in dermatitis or renal failure, primarily due to its metabolism in the liver.^1^ EG is categorized as an endocrine-disrupting hazardous chemical,^3^ associated with teratogenicity, developmental abnormalities,^4^ impaired fertility, and even testicular cancer.^3^ Acute exposure can also result in coma, seizures, central nervous dysfunction, and potentially death.^5^

EG is commonly used in electrolytes to enhance viscosity during anodic synthesis, particularly in formulations containing NH_4_F and H_2_O for the synthesis of titanium dioxide (TiO_2_) nanotubes.^6^ Its viscosity controls the dissolution kinetics and growth rate of the TiO_2_ oxide layer, providing a favorable environment for self-organization.^7–12^ However, their environmental impact raises significant concerns. When released into aquatic systems, EG exhibits a low biodegradability. During anaerobic biodegradation, it forms toxic byproducts, such as glycolic acid, oxalic acid, and formaldehyde,^13^ posing threats to aquatic life and soil ecosystems. Although nanoporous and nanotubular materials are still scarcely employed on an industrial scale, these traditional synthesis processes pose potential environmental risks. These concerns highlight the urgent need to identify and implement more eco-friendly alternatives.

To address these concerns, the greenness of alternative electrolyte compositions can be evaluated using the AGREEprep metric system.^14,15^ This tool qualitatively assesses the environmental impact of sample preparation methods using ten core criteria derived from 12 principles of green chemistry.

Recent studies have investigated various substitutes for EG, including glycerol,^16^ alginate,^17^ plant extracts, such as *Psidium guajava*,^18^ and cellulose derivatives like carboxymethyl cellulose.^19^ As one of the world’s leading producers of *Manihot esculenta*, commonly known as cassava, Brazil offers an abundant, renewable source of natural polysaccharides that can replace toxic solvents such as EG. When cassava starch granules are heated in water, gelatinization occurs, involving hydration, swelling, and the release of amylose and amylopectin.^20^ These components interact to form a three-dimensional network that traps water molecules, resulting in viscous dispersions.^21^

Colloidal dispersions of cassava starch at concentrations ranging from 20% to 28% w/v have shown promising results as an EG replacement in the synthesis of TiO_2_ nanotubes or nanoporous materials. This substitution reduces environmental impact and aligns with the Sustainable Development Goals by using biodegradable, nontoxic, and renewable resources. Moreover, this strategy supports the broader objective of developing greener nanomaterial synthesis methodologies without compromising material performance.^22^

## 2 Experimental Section

### 2.1 Electrolyte Composition

An environmentally friendly electrolyte for nanostructured TiO_2_ synthesis was prepared using gelatinized starch derived from*Manihot esculenta* (cassava) at 20% and 28% w/v, intended to replace the traditional ethylene glycol-based electrolyte (EBE). The reference electrolyte consisted of 0.75% w/w NH_4_F and 10% v/v of H_2_O in EG.^6,12^

To prepare the starch*-*based electrolyte (SBE), starch granules were dispersed in ultrapure water at 20% and 28% (w/v) and heated twice in an 800 W microwave for 30 s to promote gelatinization. After the mixture was cooled to 25 °C, the colloidal dispersion was filtered, the volume adjusted to 250 mL, and NH_4_F was added to reach a final concentration of 0.75% (w/w).

The conductivity of each electrolyte was measured using a calibrated conductometer (AKSO and AK50) at 20 and 40 °C. Relative kinematic viscosity was determined using an Ostwald capillary viscometer and expressed in g.s./cm^3^ at 25 °C (Supporting Information).

#### 2.1.1 Analytical Greenness Metrics of Electrolyte

The greenness of the sample preparation was assessed by using the AGREEprep metric system. This tool evaluates ten principles of Green Analytical Chemistry, each assigned a default weight (1–5) based on its importance. Each criterion receives a score from 0 (red) to 1 (fully green), represented in a circular diagram with segmented sections.^15^ The central circle shows the overall greenness score, while each segment reflects performance on individual criteria through color (red to green) and width (importance).^14^ Further details are available in the Supporting Information.

### 2.2 Fabrication and Characterization of TiO_2_ Films

Nanostructured TiO_2_ films were obtained by the potentiostatic anodization of commercially pure titanium (CPTi, ASTM F67). The synthesis was designed as a complete 2^3^ factorial design of experiments (DOE) with the following variables: electrolyte composition (20% and 28% w/v starch-based electrolyte, SBE), bath temperature (20 and 40 °C), and postannealing (2 °C/min, 120 min at 450 °C). CPTi samples (1.44 cm^2^) were mechanically polished (SiC, #1200 mesh), followed by anodization at 40 V for 20 min in the test electrolytes, containing 0.75% w/w of NH_4_F and 10% v/v of H_2_O.^8^ All synthesis conditions are summarized in Table 1.

**Table 1:** Experimental Design and Sample Codes for TiO_2_ Synthesis^a^

sample code	electrolyte	temperature	post-treatment
T2S20	SBE (20% w/v)	20	As-formed
T4S20	SBE (20% w/v)	40	As-formed
T2S20A	SBE (20% w/v)	20	Annealed
T4S20A	SBE (20% w/v)	40	Annealed
T2S28	SBE (28% w/v)	20	As-formed
T4S28	SBE (28% w/v)	40	As-formed
T2S28A	SBE (28% w/v)	20	Annealed
T4S28A	SBE (28% w/v)	40	Annealed
T2E	EBE	20	As-formed
T4E	EBE	40	As-formed
T2EA	EBE	20	Annealed
T4EA	EBE	40	Annealed

a The Sample’s codes were structured as follows: “T” for CPTi, followed by bath temperature (2 = 20 °C and 4 = 40 °C), “S” for starch, followed by its concentration (20 or 28% w/v), “E” for ethylene glycol, and “A” for thermal annealing.

### 2.3 Surface Characterization

Surface morphology was analyzed by field-emission scanning electron microscopy (FE-SEM, Quanta 450, FEI, Japan). Image processing was performed using “Analyze Particles” in ImageJ software.^23^ Inner pore diameters were measured from the size distribution analysis*.* Two-dimensional (2D) surface topography and arithmetic mean surface roughness (*R*_a_) were calculated from FE-SEM images using the SurfChar plugin in accordance with ISO 25178.^24^ Further details are provided in a previous work.^7^

### 2.4 Structural Characterization

X-ray diffraction (XRD) patterns were recorded by using a diffractometer (Rigaku, 600 Benchtop, Japan) with Cu Kα radiation (λ = 1.5406 Å), operating at 40 kV and 150 mA, with a continuous rate of 3°/min. Diffractograms were analyzed using PDXL software for phase identification. Raman spectroscopy was conducted using a Witec Alpha 300R spectrometer (Bruker, USA), equipped with a 532 nm laser (7×mW) and 100× objective, acquiring 60 accumulations of 1s each.

### 2.5 Electrochemical Analysis

Electrochemical measurements were performed in artificial saliva using a conventional three-electrode cell at 37 ± 1 °C, containing 0.20 g/L of K_2_HPO_4_ and 1.20 g/L of KCl. 0.33 g/L of KSCN, 0.26 g/L of Na_2_HPO_4_, 0.70 g/L of NaCl, 1.50 g/L of NaHCO_3_, 1.50 g/L of urea, and pH adjusted to 6.7 with lactic acid.^25^

Experiments were carried out using a Potentiostat/Galvanostat (EmStat 3+, PalmSens, U.S.A.) in accordance with ASTM F2129. G59. and G3.^26–28^ Nanoporous TiO_2_ electrodes (1.44 cm^2^) were used as working electrodes, platinum foil as the counterelectrode, and Ag/AgCl as the reference electrode.

Open Circuit Potential (OCP) was recorded for 30 min with a stabilization criterion of ±3 mV/min. Linear Potentiodynamic Polarization (LPP) was performed from ±0.50 V versus OCP, at a scan rate of 1 mV/s with a 2 mV potential step.

Electrochemical parameters, including corrosion potential (*E*_corr_), corrosion current density (*I*_corr_), and corrosion rate (CR), were extracted from the polarization curves using the Tafel extrapolation method in PStrace 5.8. All measurements were conducted at least in triplicate, and results were reported as mean ± standard deviation.

### 2.6 Statistical Analysis

All quantitative results were obtained in triplicate and expressed as mean ± standard deviation. The influence of synthesis parameters on morphological, structural, and electrochemical properties was assessed by Design of Experiments (DOE). Statistical significance was evaluated using analysis of variance (ANOVA) followed by Tukey’s HSD post hoc test, with a significance threshold of *p* < 0.05.

## 3 Results and Discussion

### 3.1 Assessment of Analytical Greenness

The environmental impact of EBE and SBE electrolytes was evaluated using the AGREEprep calculator, which considers ten criteria derived from the principles of Green Analytical Chemistry (Figure 1).

**Figure 1:**
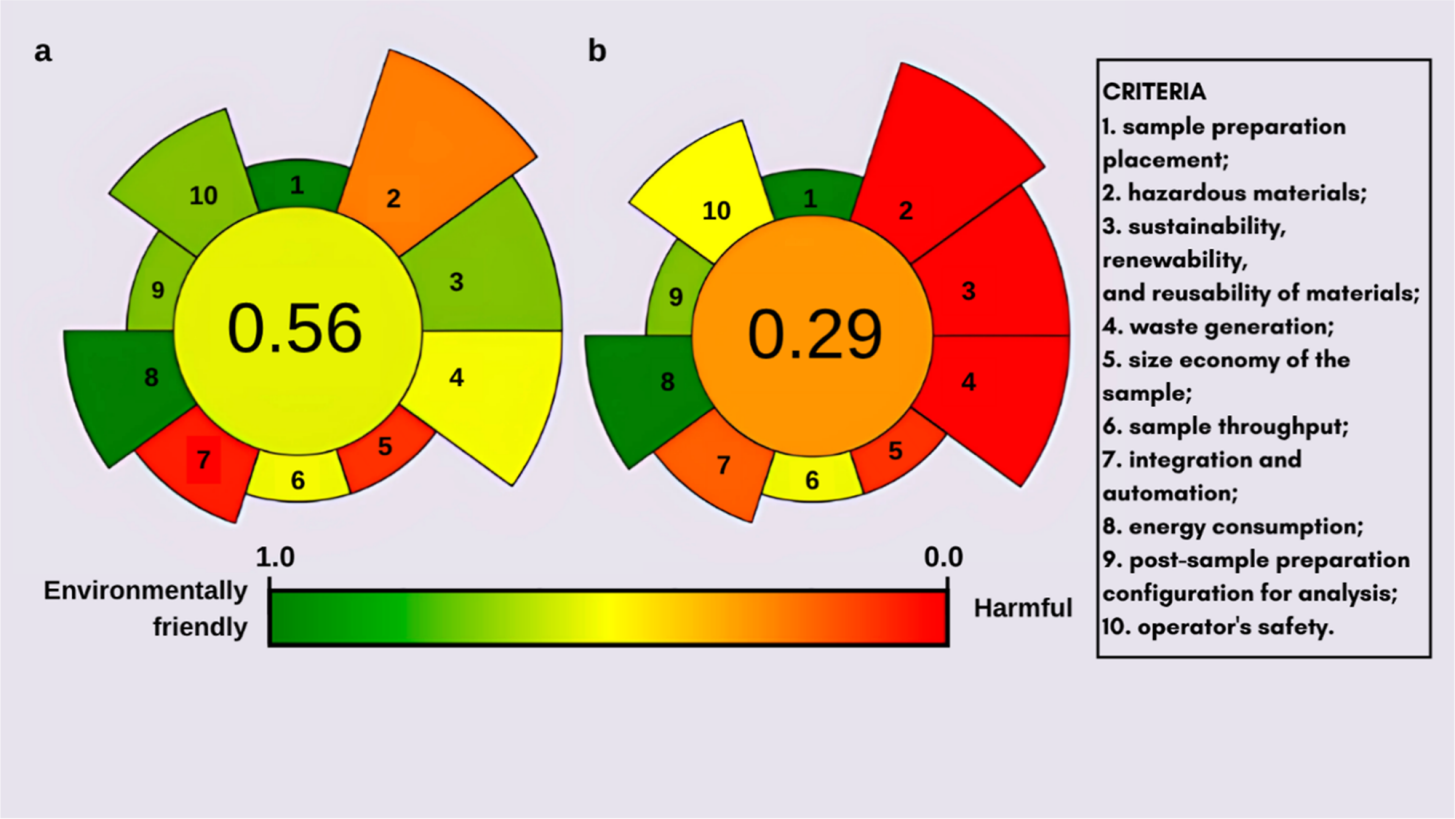
Analytical greenness metrics for sample preparation using (a) *Manihot esculenta*starch-based electrolyte (SBE) and (b) ethylene glycol-based electrolyte (EBE).

In Figure 1, the overall AGREEprep score is presented in the central circle of each diagram, summarizing each method’s sustainability index. The EBE exhibited a sustainability index (0.29) lower than that of the SBE (0.56), where values closer to 0 reflect a less sustainable process.

The SBE demonstrated superior performance in criteria 2, 3, 4, and 10, which correspond to the use of hazardous substances; proportion of sustainable and renewable materials; waste generation; and procedural safety. This advantage stems from the biodegradable, renewable, and nontoxic nature of starch, in contrast to the toxicological profile of EG.^29,30^

In contrast, criterion 7 (automation and energy efficiency) received a lower score (red shading) for SBE due to additional preparation steps including heating, cooling, filtration, and volume adjustment. Criteria 1, 5, 8, and 9, which evaluate sample transportation, sample size, energy consumption, and postpreparation sample handling, showed similar performance between the two electrolytes, as these are determined primarily by the anodization process rather than the electrolyte composition.^31–33^ The findings highlight the potential of SBE to improve the environmental performance of nanostructured TiO_2_ synthesis. Simplification of the preparation process could further enhance automation and reduce energy demand, leading to even higher AGREEprep scores.

### 3.2 Fabrication and Characterization of TiO_2_ Films

Figure 2 shows the transient current density (*j*) curves obtained during the growth of TiO_2_ films. Chronoamperometric curves show a usual three-stage pattern related to the growth of TiO_2_ nanostructures,^6^ denoted as I, II, and III.

**Figure 2:**
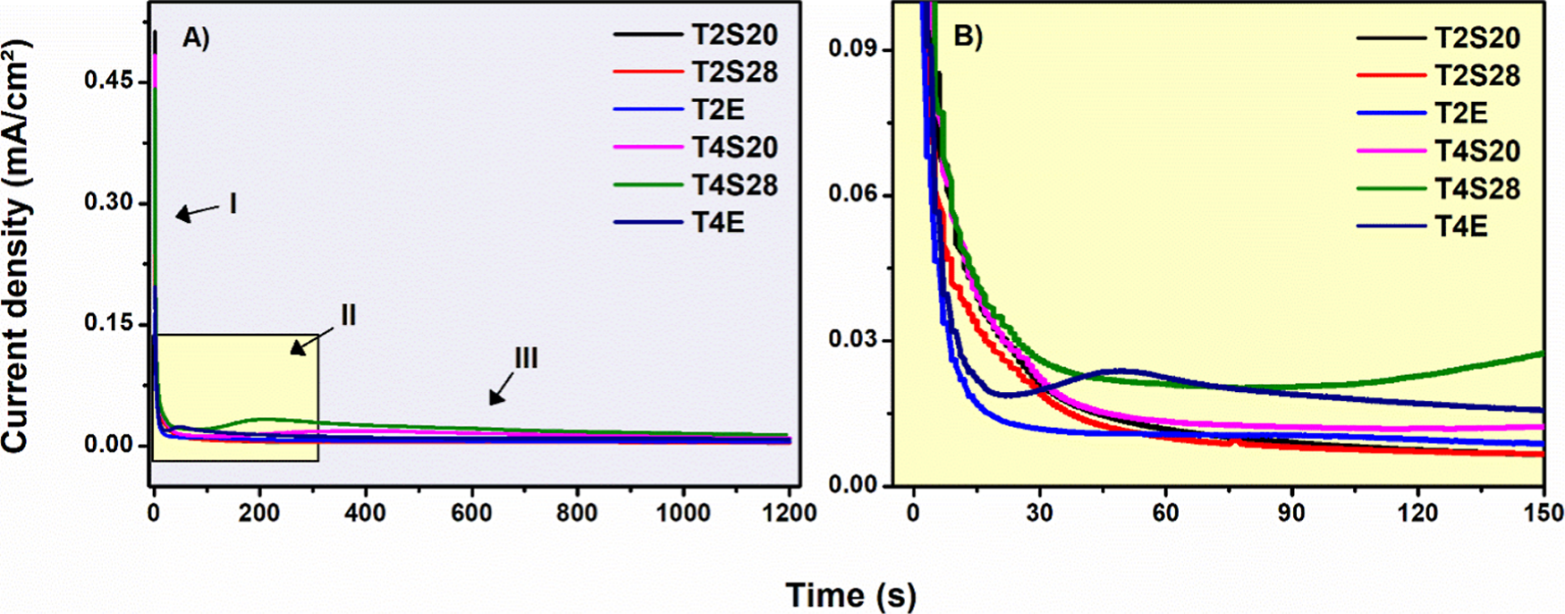
(a) Current density (*j*)-transient curves and (b) Magnified view (inset) of Stage II during the formation of anodic TiO_2_ nanostructured films.

Figure 2a details stage I: exponential *j* decay due to the formation of a compact TiO_2_ oxide layer,^34^ reaching a critical thickness, and a *j* minimum is registered.^35^ After this critical value, anions (O^2–^, OH^–^, and F^–^) penetrate this resistance oxide barrier, forming TiO_2_, Ti­(OH)_4,_ and the highly water-soluble complex [(TiF_6_)^2–^], which migrates to the bulk electrolyte solution, creating nanocavities.^36^ This implies an increase in the surface area, which increases the *j*-values and, eventually, the formation of the “nucleation peak”. After this point, the system evolves to a steady-state regime characterized by similar dissolution rates and oxide formation (stage III), wherein walls and film thicknesses evolve.^6^

For TiO_2_ nanotube arrays (TiO_2_NTs), the choice of electrolyte seems to determine not only the structural and morphological outcomes but also the physicochemical mechanisms underlying tube formation and stability. When SBE and EBE are compared, differences in parameters such as solution conductivity and dissolution pathways notably influence the degree of self-organization of the resulting nanotubes.

Regarding the electrolyte conductivity, SBE has higher ionic conductivity (11.5 and 12.5 μS/cm for 20% w/v and 9.6 and 10.6 μS/cm for 28% w/v, at 20 and 40 °C, respectively) due to the high dielectric constant of water (ε ≈ 78.5 at 25 °C) and hydrogen-bonding and ion-dipole intermolecular interactions. In contrast, EBE exhibits significantly lower conductivity (1.8 and 2.0 μS/cm at 20 and 40 °C, respectively) and a lower dielectric constant (ε ≈ 37 at 25 °C), which slows ionic mobility. However, the lower conductivity of EG-based systems results in more controlled oxide growth and self-organized structures, as shown in Figure 3.

**Figure 3:**
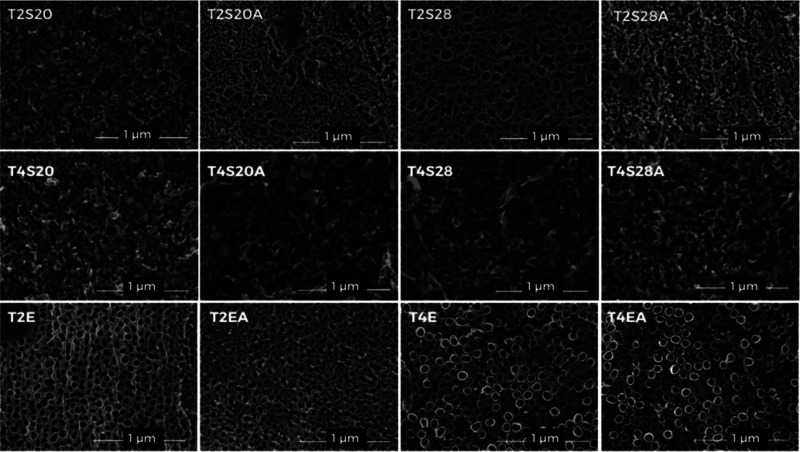
FE-SEM micrographs of TiO_2_ films obtained with SBE (indicated by S) at 20 and 28% w/v or EBE (indicated by E), according to conditions presented in Table 1.

Both electrolytes facilitate the field-assisted dissolution of [TiF_6_]^2–^, but with differences in the kinetics. Due to high ionic transport, aqueous electrolytes can promote overdissolution and disordered nanotube formation. In contrast, the reduced mobility of F^–^ and slower complexation with Ti^4+^ in EBE result in a moderated rate of dissolution and a more homogeneous morphology. During anodization, the balance between formation and dissolution rates is crucial for achieving well-aligned nanotube arrays with uniform dimensions and smooth tube-to-tube interfaces.

Figure 2b shows a temperature-dependent chronoamperometric pattern, suggesting that this variable is critical for determining anodization kinetics, particularly when eco-friendly electrolytes are used.

Films obtained at 20 °C exhibit the absence of this peak in both SBE and EBE. In contrast, at 40 °C, a distinct peak is observed for the T4E condition (electrolyte composed of EG) and the T4S28 condition (SBE electrolyte, consisting of 28% w/v of starch), suggesting favorable conditions for ordered nanotube formation, although it has been demonstrated that the nucleation peak is not necessary for the formation of nanostructures.^8–10,12^

Additionally, the polysaccharide nature of starch may introduce additional complexity. Partial degradation or oxidation during anodization can produce organic byproducts that adsorb onto the growing oxide surface, potentially interfering with tube organization or contributing to structural heterogeneity.^37–39^

The effects of the electrolyte composition and annealing can be observed in the FE-SEM images shown in Figure 3.

TiO_2_ nanoporous structures were successfully obtained under all synthesis conditions. However, as previously discussed, samples synthesized with SBE exhibited a degree of structural organization that was lower than those prepared with EBE. SBE-based samples present an interconnected, sponge-like nanoporous morphology, rather than as discrete tubes as EBE-based samples do. When the surfaces obtained by SBE were compared, T2S28 (20 °C, 28% w/v of starch, as-formed) exhibited the most ordered morphology, resembling the tubular structures obtained by EBE control. This occurs due to an association between high electrolyte viscosity (discussed in the Supporting Information) and moderate conductivity, which promotes controlled ion mobility and oxide dissolution compared to 20% SBE electrolyte.

This is particularly relevant since aligned nanopores are associated with enhanced osseointegration and bioactivity.^40,41^

Surface roughness (*R*_a_) and pore diameter were further analyzed (Figure 4). The *R*_a_ parameter was associated with a threshold for cell adhesion, orientation, differentiation, and other biological processes that occur after implant placement in the bone tissue.^7,32^

**Figure 4:**
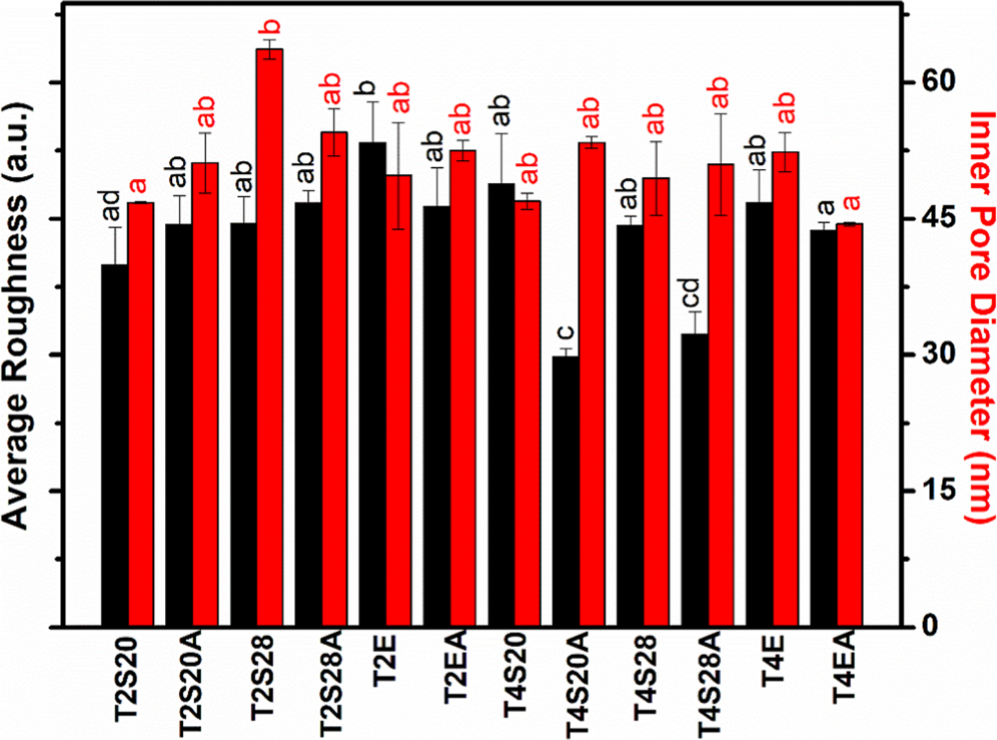
Inner pore diameter and average roughness values (*R*_a_) for TiO_2_ films. *Superscript letters (a–d) refer to results of the Tukey test. Values sharing the same letter are not statistically different (*p* > 0.05).

The results presented in Figure 4 reveal a significant variation in pore diameter (ranging from 44.39 to 63.65 nm) and in surface roughness (ranging from 29.74 to 53.37 au); ANOVA/Tukey tests confirmed statistically significant differences. The smallest pore diameters were found in T4EA and T2S20, whereas the sample obtained in SBE at 20 °C in 28% w/v (T2S28) presented the largest pores, likely due to enhanced ion transport and oxide dissolution.

Regarding roughness, T2E (EBE, 20 °C) showed the highest Ra, while T4S20A (SBE, 40 °C, 20% w/v, annealed) exhibited the lowest. Furthermore, the T4S20A sample showed roughness similar to that of the T4S28A sample, suggesting that samples synthesized with SBE at 40 °C and heat-treated generally exhibit roughness lower than that under other conditions. Samples T4S28, T2S20A, T2S28, T2EA, T2S28A, T4E, T4S20, and T2E did not present significant statistical differences.

DOE analysis revealed that neither the individual variables (temperature, SBE concentration, and heat treatment) nor their interactions significantly influenced the pore diameter. At the same time, Ra was affected by all three variables investigated: the interaction between heat treatment and synthesis temperature, as well as the combined interaction of all three variables. This result is in concordance with what is reported elsewhere.^42^

To investigate the influence of thermal annealing on the crystallinity of nanostructured TiO_2_ films, X-ray diffraction (XRD) and Raman spectroscopy analyses were carried out. As shown in Figure 5, the XRD patterns of the as-anodized samples reveal diffraction peaks corresponding to hexagonal titanium (ICSD 044390), indicating the amorphous nature of the as-formed oxide layer. After annealing at 450 °C, characteristic peaks of the anatase phase emerged at 25.3, 37.8, 48.0, 54.0, and 55.1 (2 theta), corresponding to the (101), (004), (200), (105), and (211) planes, respectively (ICSD 082084). No rutile reflections were detected, confirming the selective formation of anatase. This transition is well documented and typically occurs between 350–500 °C, depending on the anodization parameters and nanotube wall thickness.^43,44^ The anatase phase is particularly desirable for biomaterial applications due to its higher mechanical resistance and its active role in osteogenic differentiation and proliferation.^41,45^ Importantly, both SBE- and EBE-derived films exhibited similar crystallization behavior upon heat treatment, demonstrating that the choice of electrolyte did not hinder phase transformation, as seen in the Supporting Information. T4S28A presented a higher anatase percentage of all starch-based samples, similar to that of T2EA.

**Figure 5:**
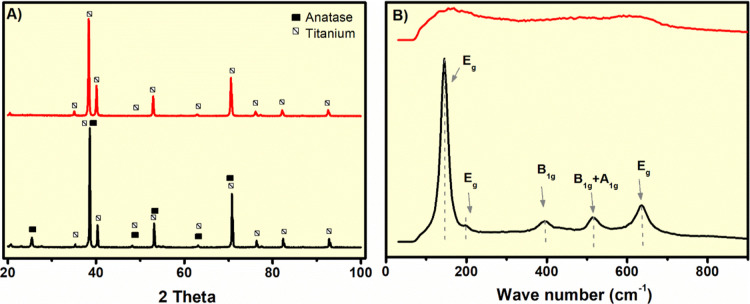
(a) XRD patterns and (b) Raman spectra of chosen TiO_2_ films before (T4S20, red lines) and after (T4S20A, black lines) annealing.

Complementary Raman spectroscopy analysis (Figure 5) corroborated the XRD findings. The untreated samples showed no characteristic vibrational bands. In contrast, samples subjected to annealing exhibited distinct anatase TiO_2_ Raman modes at approximately 144 cm^–1^ (*E*_g_), 197 cm^–1^ (*E*_g_), 400 cm^–1^ (*B*_1g_), 519 cm^–1^, (*A*_1g_ + *B*_1g_), and 640 cm^–1^ (*E*_g_), which are consistent with the anatase phase of TiO_2_.^46^

*E*_g_ modes correspond to symmetric stretching vibrations of Ti–O bonds, while the *B*_1g_ and *A*_1g_ modes are associated with symmetric and antisymmetric bending of O–Ti–O linkages, respectively.^47^ The prominent band around 144 cm^–1^ is a sensitive indicator of anatase crystallinity and grain size; its intensity and full-width at half-maximum (FWHM) can be used to estimate crystallite quality and local disorder.^48^ Extracted fwhm data are available in the Supporting Information. Although both sets of results corroborate the XRD findings, the starch-based samples showed slightly higher fwhm values due to greater local heterogeneity within the nanotube walls and a higher residual defect density.

Interestingly, the presence of a band near 639–640 cm^–1^ may also be indicative of lattice imperfections or defect states within the anatase framework.^49^ Such spectral shifts and broadening have been attributed to oxygen vacancies and local defects arising from the partial reduction of Ti^4+^ to Ti^3+^ during annealing, which alter the electronic structure, wettability, and catalytic activity of TiO_2_.

These results demonstrate that the use of starch-based electrolytes does not compromise the structural properties of the TiO_2_ films. Regardless of electrolyte composition, annealing consistently yielded the desired anatase phase with comparable crystalline and phase purity.

### 3.3 Electrochemical Analysis

Electrochemical stability is critical for assessing the long-term performance of implantable biomaterials. Upon the interaction of body fluids, deteriorative reactions could occur on the surface, including corrosion, which poses implications for long-term stability.^50^ The corrosion resistance of TiO_2_ nanotube films was assessed by open circuit potential (OCP) monitoring and linear potentiodynamic polarization (LPP) tests in artificial saliva at 37 ± 1 °C (Figure 6). Table 2 summarizes the OCP and electrochemical parameters (*E*_corr_, *I*_corr_, PR, and CR) extracted by Tafel extrapolation from LPP curves.

**Figure 6:**
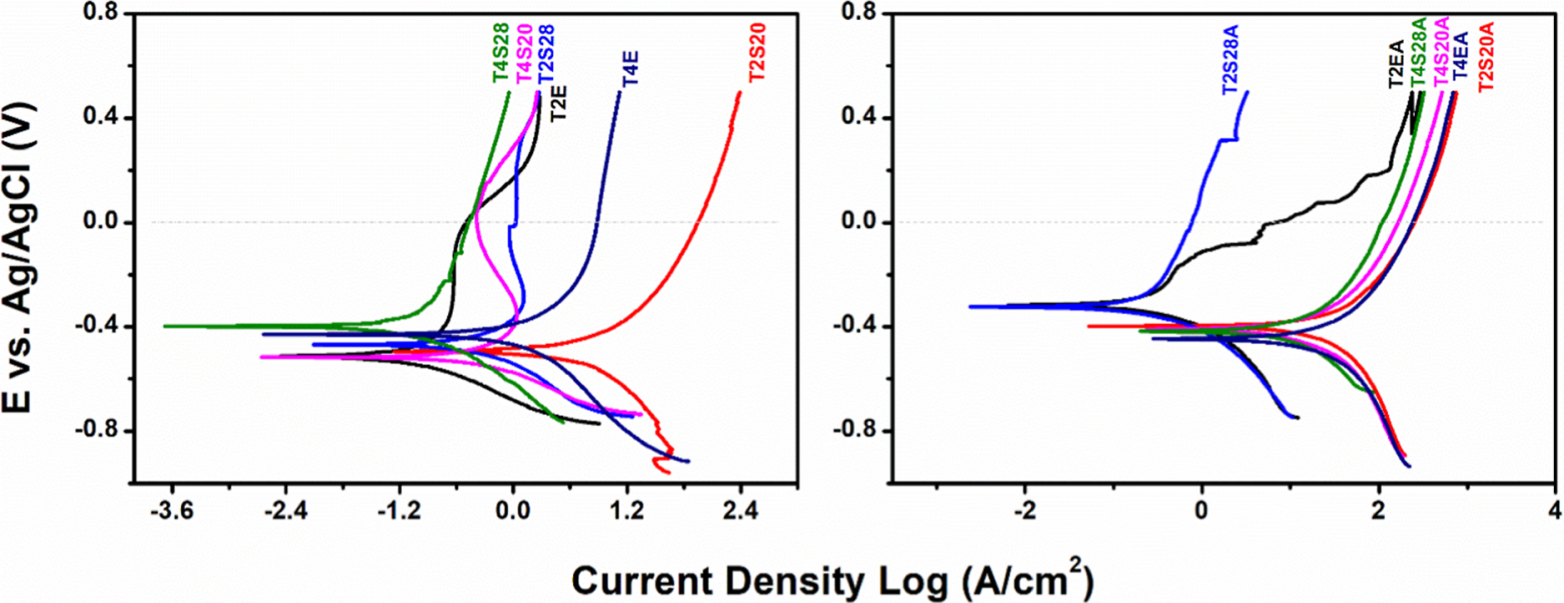
Linear Potentiodynamic Polarization curves (LPP) of nanostructured TiO_2_ samples performed in artificial saliva at 37 ± 1 °C.

**Table 2:** Electrochemical Parameters Extracted from Linear Potentiodynamic Polarization curves (LPP) Performed in Artificial Saliva at 37 ± 1 °C[Table-fn t2fn1] ^,^ [Table-fn t2fn2]

sample codes	OCP (V)	*E*_corr_ (V)	*I*_corr_ (10^–6^ A/cm^2^)	PR (10^+5^ Ω)	CR (10^–2^ mm/year)
**T2S20**	–0.22 ± 0.10^a^	–0.22 ± 0.00^a^	1.09 ± 0.25^ab^	0.99 ± 0.85^ab^	3.80 ± 0.01^ab^
**T2S20A**	–0.32 ± 0.11^a^	–0.34 ± 0.00^a^	1.11 ± 0.69^ab^	1.63 ± 1.20^ab^	3.90 ± 0.02^ab^
**T2S28**	–0.20 ± 0.06^a^	–0.25 ± 0.15^a^	0.10 ± 0.00^a^	6.98 ± 1.53^ab^	0.30 ± 0.00^a^
**T2S28A**	–0.16 ± 0.13^a^	–0.30 ± 0.03^a^	0.08 ± 0.04^a^	4.41 ± 2.32^ab^	0.25 ± 0.00^a^
**T4S20**	–0.19 ± 0.07^a^	–0.44 ± 0.08^a^	0.27 ± 0.03^a^	1.78 ± 0.77^ab^	0.95 ± 0.00^a^
**T4S20A**	–0.24 ± 0.04^a^	–0.44 ± 0.03^a^	2.16 ± 0.34^b^	0.10 ± 0.05^a^	7.50 ± 0.01^b^
**T4S28**	–0.24 ± 0.04^a^	–0.39 ± 0.05^a^	0.23 ± 0.02^a^	3.84 ± 0.22^ab^	0.80 ± 0.00^a^
**T4S28A**	–0.21 ± 0.09^a^	–0.31 ± 0.00^a^	0.44 ± 0.26^a^	2.00 ± 0.43^ab^	1.50 ± 0.01^a^
**T2E**	–0.09 ± 0.26^a^	–0.31 ± 0.21^a^	0.06 ± 0.02^a^	7.84 ± 2.46^b^	0.20 ± 0.00^a^
**T2EA**	–0.22 ± 0.02^a^	–0.28 ± 0.03^a^	0.47 ± 0.31^a^	1.12 ± 0.64^ab^	1.60 ± 0.01^a^
**T4E**	–0.29 ± 0.05^a^	–0.53 ± 0.11^a^	0.59 ± 0.21^a^	0.91 ± 0.37^ab^	2.05 ± 0.01^a^
**T4EA**	–0.23 ± 0.05^a^	–0.38 ± 0.04^a^	0.36 ± 0.19^a^	1.91 ± 1.37^ab^	1.25 ± 0.01^a^

* Superscript letters (a,b) refer to results of the Tukey test.

** Values sharing the same letter are not statistically different (*p* > 0.05).

Regarding Table 2, the OCP values did not exhibit statistically significant differences among the samples. This result can be attributed to their active electrochemical behavior, such as ongoing partial dissolution of the TiO_2_ layer and the presence of low resistance zones, which increased the number of available potential pitting sites.^51^ This suggests that, under open-circuit conditions, all samples undergo spontaneous anodic or cathodic processes, regardless of electrolyte composition or thermal treatment.

Regarding LPP, ANOVA/Tukey analysis revealed interesting trends. SBE-derived samples demonstrated that CR depends on the starch concentration and thermal treatment. Samples synthesized with 28% w/v of starch (T2S28 and T2S28A) showed lower CR parameters of 0.25–0.30 × 10^–2^ mm/year, without statistical difference from samples obtained in EBE. This is not observed with 20% w/v starch, which statistically yields samples with the highest CR. For these samples, a higher corrosion current density (*I*_corr_) can indicate the corrosion of the underlying metal substrate beneath the film/oxide layer, as discussed in a previous work.^7,52^

These results suggest that increasing the polysaccharide content in the electrolyte may modulate the dissolution kinetics and promote better self-organization of the nanotube structure, as discussed above. Organic incorporation during oxide growth seems to be negligible and does not affect CR, as cited in related literature.^7,17^ Moreover, these outcomes highlight the potential of SBE as a sustainable and biocompatible alternative to conventional EBE. Lower CR samples could also be related to the anatase crystalline phase for annealed samples, contributing to enhanced chemical passivation and reduced electron transfer activity at the interface.^53,54^

Among all samples, only T2S28, T2S28A, and T2E met the acceptable corrosion rates for metallic implants (<0.25 × 10^–3^ mm/year^55^). However, as none of the CR values showed statistical differences, all tested samples, except T4S20A, could be used as metallic implants.

In summary, the combination of electrolyte composition and thermal treatment enables the modulation of nanostructured TiO_2_ properties to achieve high surface integrity and corrosion resistance, as required for biomedical implants and biosensors. While EG-based systems remain the gold standard for producing highly ordered nanotubes with excellent stability, optimized SBE formulations represent a viable green alternative, capable of delivering comparable performance with significantly lower environmental impact.

## 4 Conclusions

This study demonstrates the successful synthesis of TiO_2_ nanoporous films using both ethylene glycol-based (EBE) and starch-based aqueous (SBE) electrolytes, focusing on their morphological, electrochemical, and surface roughness parameters. The results indicate that the use of starch, a biodegradable and environmentally friendly additive, not only enables the formation of nanostructured films but also significantly reduces the environmental impact, which was confirmed by AGREEprep greenness metrics.

Electrochemical analysis revealed distinct behaviors between EBE and SBE systems, with variations in current density profiles and nucleation peaks, suggesting differences in oxide growth kinetics and self-organization mechanisms. Postsynthesis surface roughness (*R*_a_) and inner pore diameter values varied significantly across synthesis conditions. In particular, the T2S28 sample showed better correspondence with samples anodized in EBE. Beyond electrolyte composition and synthesis temperature, thermal treatment also affects the morphology in samples anodized in SBE.

Between all samples, only T2S28, T2S28A, and T2E met the acceptable corrosion rates for metallic implants (<0.25 × 10^–3^ mm/year). However, as none of the CR values showed statistical differences, all tested samples could be used as metallic implants, except T4S20A.

The findings highlight that the choice of electrolyte and processing parameters can be strategically tuned to modulate the properties. Furthermore, starch-based systems proved to be a viable alternative to conventional organic electrolytes, offering a greener and potentially safer route for biomedical applications involving TiO_2_ films.

## Supplementary Material



## Notes

Additional experimental details and results, including viscosity, AGREEprep metric, XRD, and Raman data; electrolyte conductivity and relative kinematic viscosity data; detailed AGREEprep evaluation criteria and scoring parameters; supplementary FE-SEM image analysis; additional XRD diffractograms and phase quantification data; Raman spectra with full width at half-maximum (fwhm) analysis; and complementary statistical outputs from DOE/ANOVA (PDF)

## Abbreviations

titanium dioxide: (TiO_2_)
ethylene glycol: (EG)
ethylene glycol-based electrolytes: (EBE)
starch-based electrolyte: (SBE)
TiO_2_ nanotubes: (TiO_2_NTs)
commercially pure titanium: (CPTi)
design of experiments: (DOE)
field-emission scanning electron microscopy: (FE-SEM)
surface roughness: (*R*_a_)
X-ray diffraction: (XRD)
open circuit potential: (OCP)
linear potentiodynamic polarization: (LPP)
polarization resistance: (PR)
corrosion potential: (*E*_corr_)
corrosion current density: (*I*_corr_)
corrosion rate: (CR)
analysis of variance: (ANOVA)
current density: (*j*)
