# Supplementary material for: Starch-Based Eco-Friendly Electrolyte from Manihot esculenta for the Anodic Synthesis of Nanostructured TiO2 Films
Source: ACS Omega. 2026 Apr 30;11(18):26402–10. doi: 10.1021/acsomega.5c11864 (PMC13176993; doi:10.1021/acsomega.5c11864)
Supplement: Supplementary file 1 [file ao5c11864_si_001.pdf]

Starch-Based Eco-Friendly Electrolyte from *Manihot esculenta* for the Anodic Synthesis of Nanostructured TiO_2 Films

Isabelli C. Baradel[a], *Anna P. Simon*[a,b], *Tatiane L. C. Oldoni*[a], *Fauze J. Anaissi*[c], *Mariana S. Sikora*[a,c*]

[a]Department of Chemistry, Universidade Tecnológica Federal do Paraná (UTFPR), Campus Pato Branco – 85503-390, Via do Conhecimento, Pato Branco, Paraná, Brazil

[b]Federal Institute of Paraná (IFPR), Rodovia BR 163, 2115, Industrial, 85700-000, Barracão, Brazil.

[c]Department of Chemistry, Midwestern Paraná State University (UNICENTRO), Campus CEDETEG – 85040-167, Alameda Élio Antonio Dalla Vecchia, Guarapuava, Paraná, Brazil

***E-mail:** marianasikora@utfpr.edu.br

KEYWORDS: Nanostructured TiO_2, Green electrolytes, Starch-Based Electrolyte (SBE), Corrosion Resistance, Biomaterial.

Supporting Information

1. Viscosity analysis

The relative kinematic viscosity of the electrolytes was determined at 25 ˚C using an Ostwald capillary viscometer, expressed in mm^2/s. Electrolyte solutions were introduced into the viscometer, and the flow time between two calibrated marks was recorded in triplicate using a digital stopwatch. Kinematic viscosity was calculated using the viscometer constant, and the dynamic viscosity (η) was obtained by multiplying the kinematic viscosity by the solution density. These measurements were used to compare the flow behavior of aqueous electrolytes based on ethylene glycol and starch.

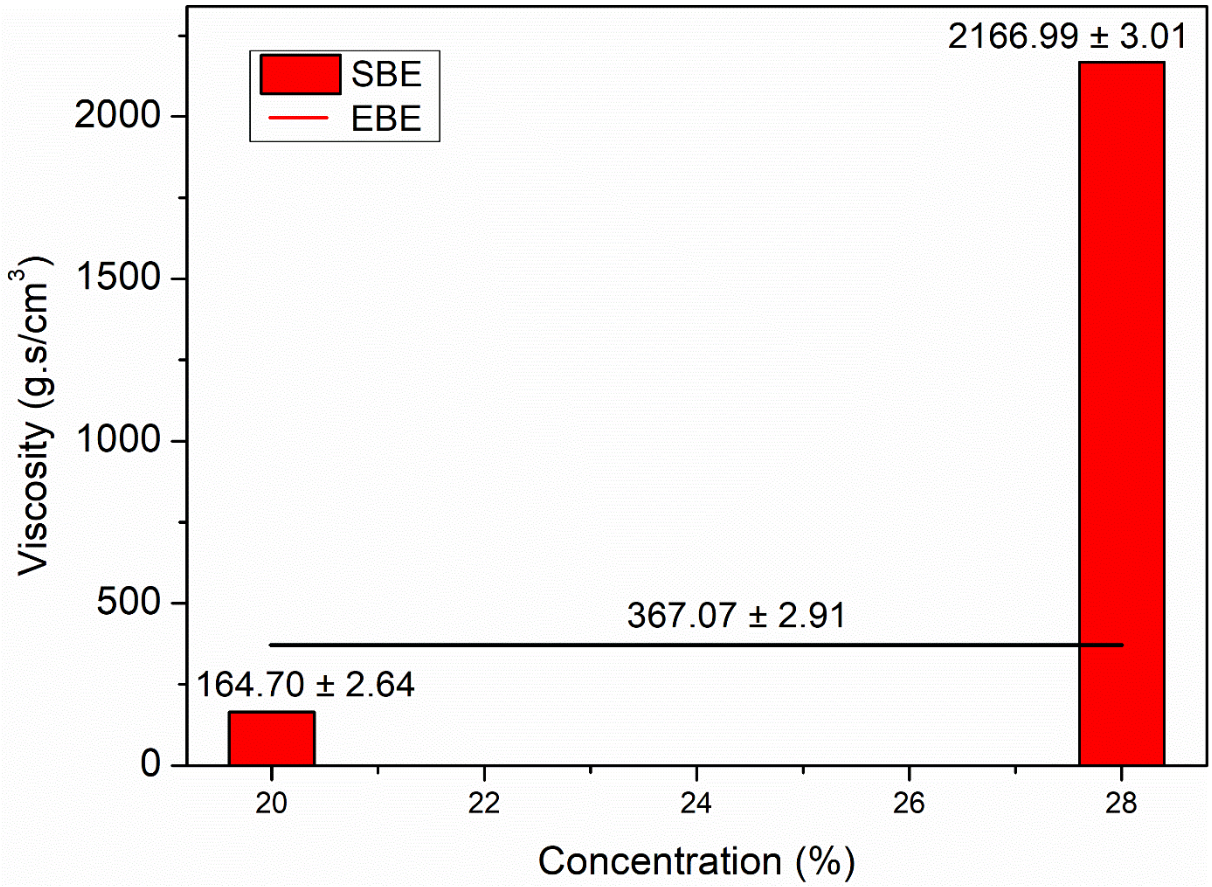

Figure S1. Viscosity of *Manihot esculenta* starch-based electrolytes as a function of concentration (20–28%), compared with the ethylene glycol control (black line).

The viscosity data presented in the graph highlights a stark contrast between the 20% and 28% *Manihot esculenta* starch-based electrolyte (SBE) and the ethylene glycol (EBE) control, represented by the horizontal black line. EBE presents a moderate and stable viscosity.

At a 20% starch concentration, the viscosity remains relatively low and is significantly lower than that of EBE. At this concentration, partial gelatinization is observed, and the electrolyte preserves the flow characteristics, which are dominated by water mobility. In contrast, at 28% starch, viscosity increases dramatically due to the formation of a three-dimensional gel network, driven by increased starch chain entanglement and gelatinization. This can reduce flow capacity and increase resistance within the electrolyte.

2. Analytical Greenness Metrics in Sample Preparation (AGREEprep metric)

The environmental performance of the electrolytes used for the anodization-based preparation of nanostructured TiO_2 was evaluated using the AGREEprep tool (Figures S2 and S3).

AGREEprep

Analytical Greenness Metric for Sample Preparation

02/12/2024 00:34:34

Report

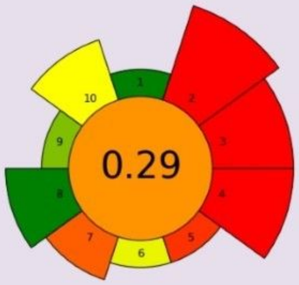

#	Criterion		Score	Weight
1.	**Sample preparation placement:**	In-line/In situ	1.00	1
2.	**Hazardous materials:**	90.818 [g or mL]	0.00	5
3.	**Sustainability, renewability, and reusability of materials:**	< 25% of reagents and materials are sustainable or renewable, but can only be used ONCE	0.00	4
4.	**Waste:**	90.818 [g or mL]	0.00	4
5.	**Size economy of the sample**	Mass or volume of the sample: 50 [g or mL]	0.10	1
6.	**Sample throughput:**	10 [samples/h]	0.54	1
7.	**Integration and automation**	Sample prep. steps: 3 steps, Manual systems	0.19	2
8.	**Energy consumption:**	0 [W]	1.00	3
9.	**Post-sample preparation configuration for analysis:**	Spectrophotometry, surface analysis techniques, voltammetry, potentiometry, etc.	0.75	1
10.	**Operator's safety:**	2 hazards	0.50	3

Figure S2. Analytical greenness assessment (AGREEprep metric) for TiO_2 nanotube synthesis using an organo-aqueous electrolyte, containing ethylene glycol (EBE).

AGREEprep

Analytical Greenness Metric for Sample Preparation

02/12/2024 00:31:47

Report

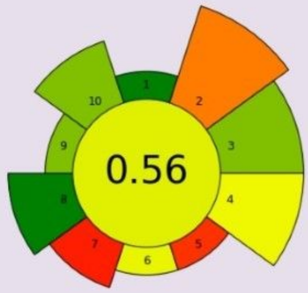

#	Criterion		Score	Weight
1.	Sample preparation placement:	In-line/In situ	1.00	1
2.	Hazardous materials:	1.858 [g or mL]	0.24	5
3.	Sustainability, renewability, and reusability of materials:	> 75% of reagents and materials are sustainable or renewable	0.75	4
4.	Waste:	1.858 [g or mL]	0.53	4
5.	Size economy of the sample	Mass or volume of the sample: 50 [g or mL]	0.10	1
6.	Sample throughput:	10 [samples/h]	0.54	1
7.	Integration and automation	Sample prep. steps: 5 steps, Manual systems	0.06	2
8.	Energy consumption:	1 [W]	1.00	3
9.	Post-sample preparation configuration for analysis:	Spectrophotometry, surface analysis techniques, voltammetry, potentiometry, etc.	0.75	1
10.	Operator's safety:	1 hazard	0.75	3

Figure S3. Analytical greenness assessment (AGREEprep metric) for TiO_2 nanotube synthesis using a starch-based electrolyte (SBE).

Comparison of the results highlights significant differences in sustainability. The SBE achieved a higher overall score (0.56), attributed to the use of renewable reagents, lower toxicity, and

reduced energy demand. In contrast, the EBE method presented a substantially lower score (0.29), primarily due to the use of hazardous, non-renewable reagents and greater waste generation. Although EE offered slightly higher automation and energy efficiency, its overall sustainability was compromised by increased environmental and health risks. These results reinforce the importance of incorporating greenness metrics, such as AGREEprep, into nanomaterial development to ensure a balance between performance, safety, and environmental responsibility.

3. Crystallographic and Vibrational Characterization of TiO_2 Nanotubes

Structural properties of nanostructured TiO_2 films were characterized by X-ray Diffraction (XRD) and Raman spectroscopy for all samples (Figures S4 and S5). Values of anatase area extracted are presented in Table S1.

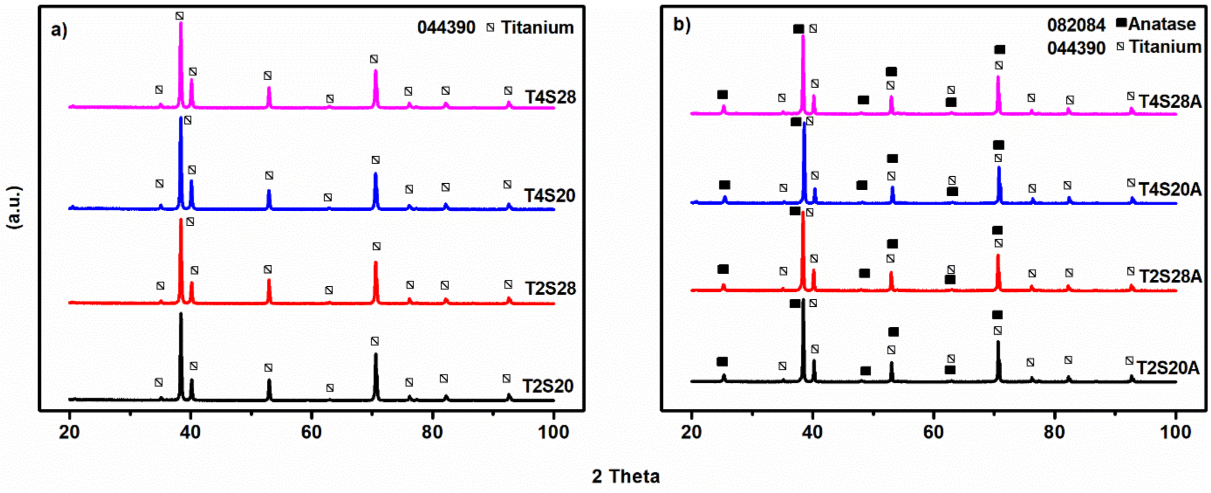

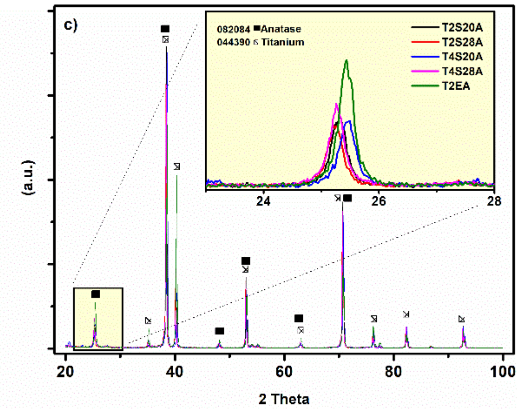

Figure S4. XRD patterns of nanostructured TiO_2 samples: a) as-formed, b) after annealing at 450 ˚C, and c) zoom at the anatase region.

Table S1. Anatase percentage (%A) extracted by XRD.

ID	%A
T2S20A	10.43
T2S28A	8.74
T4S20A	8.92
T4S28A	9.66
T2EA	8.89

Figure S5 presents the Raman spectra of nanostructured TiO_2 samples.

Figure S5. Raman spectra of nanostructured TiO_2 samples, a) before and b) after annealing.

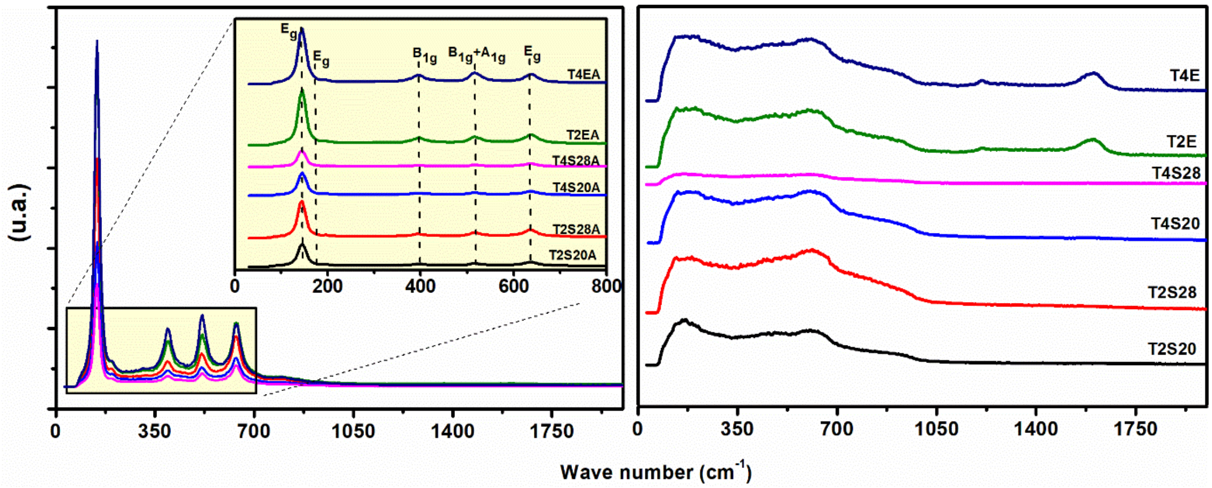

The as-formed samples exhibited amorphous patterns with no distinct diffraction peaks. After annealing, the characteristic anatase phase reflections were clearly identified by XRD, corroborated by Raman spectra showing the Eg and B1g vibrational modes typical of anatase. These results confirm successful crystallization and demonstrate that both electrolytes yielded TiO_2 with comparable structural features after thermal treatment.

Table S2. Full width at half maximum (FWHM) of the Eg mode (~144 cm^{-1}) from the Raman spectra

ID	FWHM (cm^{-1})
T2S20A	7.59 ± 1.33
T2S28A	9.04 ± 0.98
T4S20A	7.08 ± 1.39
T4S28A	9.48 ± 1.08
T2EA	8.66 ± 0.54
